# The impact of implantable cardioverter defibrillator on the prognosis of nonischemic dilated cardiomyopathy patients compared with standard medical treatments

**DOI:** 10.1002/clc.24022

**Published:** 2023-04-14

**Authors:** Hossein Salehi Omran, Farah Naghashzadeh, Rana Irilouzadian, Sina Dolatshahi, Mohammad Taghi Hedayati Goudarzi, Mohammad Taghi Salehi Omran

**Affiliations:** ^1^ School of Medicine Shahid Beheshti University of Medical Science Tehran Iran; ^2^ Lung Transplantation Research Center, National Research Institute of Tuberculosis and Lung diseases (NRITLD) Shahid Beheshti University of Medical Sciences Tehran Iran; ^3^ Burn Research Center Iran University of Medical Sciences Tehran Iran; ^4^ Cardiology Department, Rohani Hospital, School of Medicine Babol University of Medical Sciences Babol Iran; ^5^ Department of Cardiology Babol University of Medical Sciences Babol Iran

**Keywords:** implantable cardioverter defibrillator, medical therapy, nonischemic dilated cardiomyopathy, sudden cardiac death, survival

## Abstract

**Background:**

Patients with nonischemic dilated cardiomyopathy (DCM) are susceptible to arrhythmias and implantable cardioverter defibrillator (ICD) in addition to medical treatments may help prevent sudden cardiac death (SCD) and improve survival in this population.

**Hypothesis:**

We aim to investigate the impact of ICD insertion on survival and prognosis of patients with nonischemic DCM.

**Methods:**

We retrospectively analyzed data from patients with nonischemic DCM treated with medical therapy with or without ICD who referred to our hospital from January 2020 to November 2021. Patients were divided based on the treatment that they had received into two equal groups. Different variables including demographic features, comorbidities, medical treatments, hospitalization rate, function class, and left ventricular ejection fraction before and after treatments were investigated in this study. In addition, variables in survival including overall survival (OS) and SCD were compared between the two groups.

**Results:**

A total of 120 patients were investigated in this study. Mean ± SD of age and follow‐up time of patients were 64.0 ± 12.7 years old and 61.2 ± 15.9 months, respectively. Ten (16.7%) patients with medical therapy, and seven (11.7%) patients with ICD and medical therapy died during the follow‐up period (*p* = 0.25). However, the two groups had a significant difference regarding SCD (11.7% vs. 1.7%, *p* = 0.02).

**Conclusion:**

In patients with nonischemic DCM who had undergone ICD insertion in addition to standard medical treatments, SCD was significantly reduced compared with patients receiving just medical treatments. OS had no significant difference between our two studied groups.

## Graphical abstract

In a retrospective cohort study, we analyzed data from 120 participants with nonischemic dilated cardiomyopathy (DCM) treated with standard medical therapy with or without implantable cardioverter defibrillator (ICD). Patients were divided into two equal groups based on the kind of treatment they had received.

- ICD insertion significantly decreased cardiac arrhythmia leading to sudden cardiac death in patients with nonischemic DCM who were receiving standard medical treatments.
- On the other hand, ICD implantation had no significant advantage in other investigated clinical variables including overall survival (OS), hospitalization rate, and cardiac function implied by left ventricular ejection fraction and New York Heart Association function class.

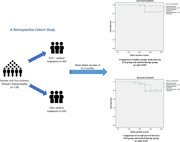

## 1 INTRODUCTION

Dilated cardiomyopathy (DCM) is considered as one of the common causes of heart failure with general prevalence of one case per 250 individuals.^1^ In addition, with annual mortality rate of 25%–30% and 5‐year mortality rate of 50%, it is recognized among the most important causes of fatality and a burden on global health system.^2^

Patients with DCM are prone to arrhythmias leading to sudden cardiac death (SCD) in about 30% of cases.^3^ Medical treatments such as beta‐blockers are recognized as influential and potential agents which can increase survival and cardiac function of patients with DCM.^4^, ^5^ Moreover, they might prevent SCD by reducing the possibility of arrhythmias.^6^ However, according to current guidelines, using implantable cardioverter defibrillators (ICDs) in addition to medical treatments is recommended for selected DCM patients to prevent sudden cardiac arrhythmias.^7^ Mortality advantages of ICD insertion are mostly recognized in patients with ischemic DCM. However, its benefits in nonischemic DCM have remained controversial.^8^

Despite coronary heart disease being generally considered as the main cause of SCD, up to 20% of cases of SCD is due to nonischemic cardiomyopathies that lead to cardiac arrhythmias.^9^

Although few articles have been published regarding survival outcomes of ICD insertion in patients with nonischemic DCM recently, their findings regarding overall survival (OS) and SCD were conflicting.^10^, ^11^, ^12^ In this study, we aim to investigate whether ICD insertion was effective for increasing OS and prognosis of patients with nonischemic DCM who underwent medical treatments in a retrospective cohort study.

## 2 MATERIALS AND METHODS

We retrospectively analyzed data from 120 patients with nonischemic DCM treated with medical therapies with or without ICD insertion who referred to Masih Daneshvari Hospital from January 2020 to November 2021.

All patients with all of the following criteria are included in this study:

1. Nonischemic DCM patients with the age between 21 and 85 years old, who were on the full tolerable guideline‐directed medical treatment
2. Left ventricular ejection fraction (LVEF) ≤ 35% as confirmed by transthoracic echocardiography (Vivid 6 echo machine, using two‐dimensional and Simpson methods).
3. Symptoms of heart failure and New York Heart Association (NYHA) functional class (FC) of II to IV.
4. Evidence of DCM confirmed by echocardiography.

On the other hand, all of the patients with a history of coronary artery disease, congenital heart failure, and acute myocarditis, confirmed by coronary angiography or stress imaging study were excluded from our article.

Patients were divided into two groups based on the kind of therapies they had received. The first group consisted of the patients that were candidates for ICD and cardiac synchronization therapy (CRT) but declined the procedure for nonmedical reasons and received only medical treatment. The second group were ICD candidates whom device was implanted for.

Both groups had received the following treatments: angiotensin‐converting enzyme (ACE) inhibitors or angiotensin receptor blockers (ARBs), beta‐blockers (such as carvedilol and metoprolol), diuretics (such as thiazide and furosemide), aldosterone antagonists, and digoxin in those patients who did not have contraindication. All patients had received medical treatment or ICD insertion for at least 1 year before the study.

The sample size was determined based on the 5‐year lifespan of 50% for nonischemic DCM patients receiving medical treatment and 75% for nonischemic DCM patients receiving ICD and CRT, resulting in 60 patients for each group.

Complications such as pneumothorax, hemothorax, cardiac tamponade, and mild bleeding during ICD implantation were rarely observed which necessary treatment measures were taken. Other complications such as thrombosis, infection, and displacement of the leads were also observed and were adequately treated. No deaths related to ICD insertion were reported.

Patients were followed up every 3 months for evaluation of heart failure progression, vital signs, mortality, sudden death rate, and arrhythmias for at least 1 year.

Different variables including demographic features, underlying diseases, medical treatments, hospitalization rate, and NYHA FC and LVEF before and after treatments were evaluated in this study. In addition, variables in survival including OS and SCD were compared between the two groups.

SCD was defined as mortalities that were reported due to cardiac arrhythmia and OS was considered as mortalities happened due to all causes including cerebral emboli, severe heart failure, and pulmonary edema.

### 2.1 Statistical analysis

Data analysis was performed by IBM SPSS Statistics for Windows, version 23 (IBM Corp). The *p* value less than 0.05 was considered statistically significant. Mean and standard deviation (SD) were used to express the quantitative data, while frequency and percentage were used to express the qualitative data. The assumption of normality was checked by using Kolmogorov–Simonov test. Qualitative data were evaluated by *χ* ^2^ test and quantitative variables were compared by *t* test or Mann–Whitney *U* test. We examined the possible role of different variables in survival by using Kaplan–Meier test.

## 3 RESULTS

One hundred and twenty patients were included in this study which half of them had received only medical treatments and the other half had received medical treatment with ICD insertion, referred to as ICD group. Mean ± SD (range) age of participants was 64.0 ± 12.7 (22–83) years old. A total of 86 individuals (71.7%) were men and 34 (28.3%) were women. Mean ± SD (range) of follow‐up duration of patients was 61.2 ± 15.9 (24–96) months. Comparison of different baseline characteristics between the two groups is illustrated in Table 1. Most of our patients had NYHA FC III in both groups. Patients' FC and LVEF before initiating treatment were not significantly different between the two groups. Also, no significant difference regarding underlying diseases was observed between the two studied groups at the beginning of the diagnosis (Table 1).

**Table 1:** Comparison of different baseline variables between ICD group and medical therapy group.

Variables			Medical therapy group (*n* = 60)	ICD group (*n* = 60)	*p* Value
Age, years			65.6 ± 12.1	62.4 ± 13.3	0.21
Gender		Male	42 (70.0%)	44 (73.3%)	0.69
		Female	18 (30.0%)	16 (26.7%)	
DM		Yes	14 (23.3%)	14 (23.3%)	1.00
		No	46 (76.7%)	46 (76.7%)	
HTN		Yes	33 (55.0%)	29 (48.3%)	0.47
		No	27 (45.0%)	31 (51.7%)	
DLP		Yes	28 (46.7%)	21 (35.0%)	0.19
		No	32 (53.3%)	39 (65.0%)	
CKD		Yes	6 (10.0%)	5 (8.3%)	0.75
		No	54 (90.0%)	55 (91.7%)	
Prior NYHA FC		**FC II**	10 (16.7%)	15 (25.0%)	0.53
		FC III	36 (60.0%)	32 (53.3%)	
		FC IV	14 (23.3%)	13 (21.7%)	
Previously‐measured LVEF (%)			25.3 ± 5.3	24.6 ± 4.3	0.30
Follow‐up time, months			59.4 ± 17.2	63.0 ± 14.4	0.11

*Note*: All data are expressed as mean ± SD for quantitative variables or frequency (%) for quantitative data.

Abbreviations: CKD, chronic kidney disease; DLP, dyslipidemia; DM, diabetes mellitus; FC, function class; HTN, hypertension; ICD, implantable cardioverter defibrillator; LVEF, left ventricular ejection fraction.

Table 2 compares the types of medications that the patients were receiving. No significant difference was observed between the two groups in terms of medications including ACE inhibitors, ARBs, beta‐blockers (such as carvedilol and metoprolol), diuretics such as thiazide, furosemide, spironolactone, and digoxin.

**Table 2:** Comparison of different medications consumed by patients between ICD group and medical therapy group.

Medications		Total (*n* = 120)	Medical therapy group (*n* = 60)	ICD group (*n* = 60)	*p* Value
Beta‐blockers	Carvedilol	69 (57.5%)	35 (58.3%)	34 (56.7%)	0.81
	Metoprolol	23 (19.1%)	13 (21.7%)	10 (16.7%)	
	Others	7 (5.8%)	3 (5.0%)	4 (6.6%)	
	Total	99 (82.5%)	51 (85.0%)	48 (80.0%)	
ACE‐inhibitors		102 (85.0%)	52 (86.7%)	50 (83.3%)	0.61
ARBs		13 (10.8%)	5 (8.3%)	8 (13.3%)	0.38
Spironolactone		84 (70.0%)	44 (73.3%)	40 (66.7%)	0.43
Diuretics		103 (85.8%)	51 (85.0%)	52 (86.7%)	0.80
Digoxin		49 (40.8%)	25 (41.7%)	24 (40.0%)	0.85
Nitrate		13 (10.8%)	8 (13.3%)	5 (8.3%)	0.38

*Note*: Data are expressed as frequency (%) for variables.

Abbreviations: ACE, angiotensin‐converting enzyme; ARB, angiotensin receptor blocker; ICD, implantable cardioverter defibrillator.

Different clinical variables consisting of survival and functional factors were compared between our two groups and is demonstrated in Table 3. In the medical therapy group, 10 patients (16.7%) and in ICD group, 7 patients (11.7%) died during the follow‐up (*p* = 0.25). Most of the patients had FC II after initiating treatment in both groups. There was no significant difference between the two groups in terms of patients' LVEF and activity tolerance implied by FC after receiving treatment and the rate of hospitalizations.

**Table 3:** Comparison of different clinical outcomes between ICD group and medical therapy group.

Variables		Medical therapy group (*n* = 60)	ICD group (*n* = 60)	*p* Value
OS		50 (83.3%)	53 (88.3%)	0.25
SCD		7 (11.7%)	1 (1.7%)	0.02
Posttreatment NYHA FC	FC 1	6 (10.0%)	4 (6.7%)	0.84
	FC 2	43 (71.7%)	47 (78.3%)	
	FC 3	9 (15.0%)	7 (11.7%)	
	FC 4	2 (3.3%)	2 (3.3%)	
Posttreatment LVEF (%)		22.3 ± 4.4	22.5 ± 5.2	0.96
Hospitalization rate		1.1 ± 0.9	0.9 ± 1.0	0.20

*Note*: Data are expressed as frequency (%) for the variables.

Abbreviations: FC, function class; ICD, implantable cardioverter defibrillator; LVEF, left ventricular ejection fraction; OS, overall survival; SCD, sudden cardiac death;

While one of the 7 (1.7%) deaths among ICD group was due to SCD, 7 of 10 (11.7%) deaths from the medical therapy group was due to SCD during the follow‐up period (*p* = 0.02).

**Figure 1:**
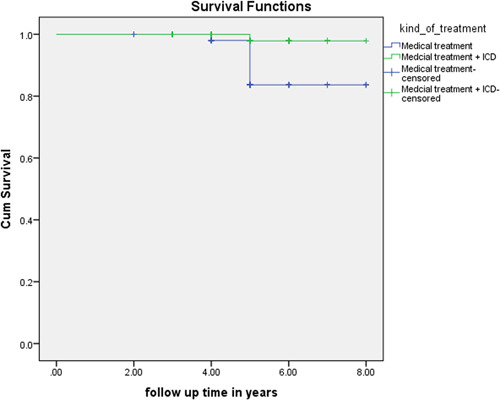
Comparison of sudden cardiac death between ICD group and medical therapy group. ICD, implantable cardioverter defibrillator.

**Figure 2:**
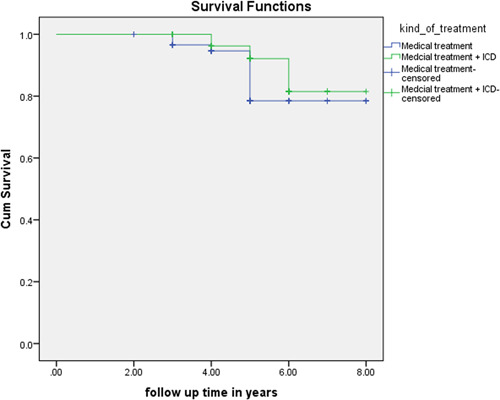
Comparison of overall survival between ICD group and medical therapy group. ICD, implantable cardioverter defibrillator

In Figures 1 and 2, survival rate of medical therapy group and ICD group is illustrated in terms of OS and SCD. OS had no significant difference between the two groups, while SCD was significantly different between them.

## 4 DISCUSSION

We aimed to compare clinical variables of potential interest including survival, function class, LVEF, and hospitalization rate between ICD group and medical therapy group. Our findings showed that there was no significant benefit of ICD implantation in patients with nonischemic DCM who underwent medical treatments in terms of all clinical variables except SCD (*p* = 0.02).

Several articles have been published regarding clinical outcomes of ICD insertion in patients with cardiomyopathy; survival outcomes of using ICD in patients with ischemic cardiomyopathy is well‐established. In a study by Moss et al.,^8^ 1232 participants with myocardial infraction before left ventricle dysfunction were included and demonstrated a 31% reduction in the mortality risk of the defibrillator group compared with patients with conventional treatment. Their results recommended ICD insertion for patients with ischemic cardiomyopathy. Also, heart failure management guidelines recently published by the American College of Cardiology/American Heart Association considers ICD insertion in patients with nonischemic cardiomyopathy with FC class of II and III, LVEF of less than 35%, and expected survival of greater than 1 year.^13^

However, there is still a controversy about ICD insertion in terms of survival and SCD prevention in patients with nonischemic DCM. In our study, we revealed that ICD implantation had significant benefit in prevention of SCD occurrence in patients with nonischemic DCM who also received medical treatments (*p* = 0.02), but no statistically difference was observed in other clinical variables including OS, posttreatment FC and LVEF, and hospitalization rate.

In a study by Gutman et al.,^10^ 452 patients with nonischemic cardiomyopathy with median follow‐up of 37.9 months were investigated regarding survival outcomes of ICD implantation. According to their findings, insertion of ICD in patients with nonischemic cardiomyopathy who had left ventricular scar reduced the mortality rate compared with standard medical therapy. In a trial with 104 patients with recent‐onset DCM and LV dysfunction which was conducted by Bansch et al. in 1990s,^14^ ICD insertion did not have any survival benefit; the cumulative survival after 6 years was 73% and 68% in the ICD and control groups, respectively. Similarly, in our study, OS was 88.3% and 83.3% in ICD group and control group, respectively (*p* = 0.25). However, we revealed that SCD was significantly lower in ICD group (11.7% vs. 1.7%, *p* = 0.02).

In a clinical trial conducted by Kober et al.^11^ in 556 patients with symptomatic, nonischemic systolic heart failure, all‐cause mortality was not significantly different in the group who underwent ICD insertion compared with group with usual treatment. However, SCD due to cardiac arrhythmia was significantly lower in patients with ICD implantation which was similar to our findings. The results of a study by Kadish et al.^15^ revealed that in patients with severe nonischemic DCM who underwent ICD with ACE inhibitors and β‐blockers, the risk of SCD were significantly lower than those who received conventional therapy with only medical treatments. However, all‐cause mortality was not significantly different between the two groups, similar to our study. Moreover, other clinical outcomes like hospitalization rate, and posttreatment FC and LVEF were also investigated in our study; no significant difference was observed regarding these variables in the two groups.

In another trial by Bardy et al.,^12^ survival outcomes of amiodarone and ICD implantation were investigated in nonischemic DCM patients. Unlike amiodarone, ICD insertion with standard medical treatments, reduced the all‐cause mortality significantly compared with conventional therapies. Contrary to our results, Strickberger et al. study^16^ in patients with nonischemic DCM and nonsustained ventricular tachycardia demonstrated that ICD insertion had no beneficial effect on survival variables including all‐cause mortality and SCD compared with the group that consumed amiodarone; however, the control group of our study did not receive amiodarone.

Our study had several strengths; first, we have examined clinical variables such as hospitalization rate, LVEF, and function class of our patients at the end of our follow‐up period in our study which was not investigated before to the best of our knowledge. In addition, this study was conducted in Iran, where few studies have been conducted on the use of ICD due to limitation in ICD insertion resources.

We also had some limitations in this study; The most important one is the limited number of patients who had undergone ICD insertion. Also, this is a retrospective study which has the limitations related to the study design. Some of our patients were candidates for ICD at the time of diagnosis but they were unwilling to receive ICD; as a result, they had only received medical treatments. None of the nonischemic DCM patients in our study was receiving amiodarone and we could not compare this drug with ICD, which is controversial topic in DCM patients. Therefore, further trial studies are warranted to validate our results. Taken all this together, risk stratification is required to determine the nonischemic DCM patients that may benefit from ICD implantation.

## 5 CONCLUSION

Our study revealed that ICD implantation significantly reduced cardiac arrhythmia leading to SCD in patients with nonischemic DCM who were receiving standard medical treatments. ICD insertion had no significant benefit in other investigated clinical variables including OS, hospitalization rate, and cardiac function implied by LVEF and NYHA FC.

## CONFLICT OF INTEREST STATEMENT

The authors declare no conflict of interest.

## Data Availability

The data that support the findings of this study are available on request from the corresponding author.
